# Reliability and Criterion Validity of Physio Master Application for the Measurement of Cervical Range of Motion in Healthy Individuals

**DOI:** 10.7759/cureus.73723

**Published:** 2024-11-15

**Authors:** Meghna Wadhwa, Mahek Panchwan, Ranganathan Arunmozhi, Vishal Verma, Shashi Singh

**Affiliations:** 1 Physiotherapy, Sardar Bhagwan Singh University, Dehradun, IND

**Keywords:** cervical range of motion, digital goniometer, physio master application, reliability, validity

## Abstract

Objective: Recent advances in smartphone applications for measuring range of motion have led to the development of the physio master application by Trinus Laboratory, Navi Mumbai. This app, equipped with a magnetometer and accelerometer, accurately measures the angle between starting and ending device positions. This study aimed to assess the inter-rater and intra-rater reliability, as well as the criterion validity, of the physio master application in measuring cervical range of motion in healthy individuals.

Method: This study included 90 healthy individuals. The two therapists measured the cervical range of motion of each participant twice by using the physio master application (for the inter-rater and intra-rater reliability) and once with the digital goniometer (for the estimation of criterion validity). Then the reliability and validity were estimated by using the intraclass correlation coefficient (ICC).

Result: The study found excellent intra-rater reliability for both therapists (ICCs: 0.77-0.96), with extension showing the highest reliability. Inter-rater reliability was also excellent (ICCs: 0.86-0.96), and criterion validity compared to a digital goniometer ranged from (0.79-0.93), with extension again showing the highest validity.

Conclusion: The results demonstrated excellent intra-rater and inter-rater reliability, along with outstanding validity when compared to a digital goniometer. These findings indicate that the physio master application is a reliable and valid alternative to traditional goniometers for measuring cervical range of motion, providing a convenient and advanced tool for clinicians and researchers.

## Introduction

Cervical spine diseases have become more common as a result of recent lifestyle changes. Approximately 30% to 50% of the population is affected by this illness on average. Women experience a far higher prevalence of illnesses than males do, with rates of 27.2% and 17.4%, respectively [1]. They are a leading contributor to morbidity, economic hardship, and disability globally. The analysis of neck ranges serves as the foundation for the American Medical Association's (AMA) functional model guidelines for assessing permanent impairment [2]. Assessing joint range during both static and dynamic, passive, and active movements is essential for musculoskeletal evaluations. This process helps in studying joint functionality, identifying imbalances, and measuring the effectiveness of treatments as a dependable outcome indicator [3].

The digital goniometer (DG) is an instrument that is being used for measuring joint ranges, particularly for static ROM, because of its affordability, ease of use, and respectable levels of validity and reliability. A study by Carey et al. showed that as an instrument for evaluating various joint angles, the DG demonstrated adequate concurrent criterion-oriented validation and comparable inter and intra-observer concordance to the universal goniometer (UG) [4]. Alternative to these hand-held goniometers a new measurement approach for measuring joint angles is smartphone goniometric apps which are proving to be consistent, economical, and efficient. There are currently several goniometric apps for phones that use various mechanisms to calculate joint angles. Cell phones can now assess joint range of motion thanks to advancements in software and app development as well as the widespread adoption of cell phones [5]. The motion sensor, gyroscope, and magnetometer that are built into smartphones provide them with the tools to measure displacements and angles [6]. Through the utilization of smartphone apps, these measures can be converted into valuable assessment information, including joint ROM [7]. Therefore, the development of smartphone apps offers clinicians new tools to use in their work, particularly for some of the more challenging joint ROMs to measure [7]. The validity, precision, and repeatability of the measures made using these applications have all been investigated, but there is not any solid proof to back up the use of phone applications to assess neck ranges [8,9].

The physio master application developed by Trinus Laboratory, Navi Mumbai, is one such application. It is an easy-to-use application that can be utilized to analyze ranges of all the joints of the body and is available for both Android and iOS operating systems. It is a very effective tool for increasing efficiency in performing a variety of assessments such as posture analysis, angle measurement, functional movement system (FMS) analysis, motion analysis, and walk test. It can store data separately for each patient that can be used as a database. Aiming to assess the physio master application's intra- and inter-rater reliability in evaluating the criteria validity of cervical ranges using a DG as the reference standard, this study considers potential applications of smartphones in rehabilitation as well as the positive outcomes achieved with DGs.

## Materials and methods

Study design

Reliability between and within raters was employed in a descriptive correlation strategy to assess the physio master application's reliability. Additionally, we evaluated concrete validity using the DG as the reference standard to assess the validity of this application. Physio master application's reliability and criterion validity have not been previously studied; hence the participants in this study were individuals who were free of cervical issues or neck pain.

Participants

Ninety-nine healthy volunteers, ages 16 to 30, comprised our heterogeneous sample. Individuals who satisfied the requirements for inclusion were at least 15 years old and did not have any neck pain or any known problems with their cervical spine. Individuals with neurological, autoimmune, and psychological conditions such as dementia, forgetfulness, delirium, or any known cervical pathology within the previous year were not allowed to participate. Convenient sampling was used to choose the population, and each subject gave their permission to participate in the research. They received no payment or other form of reimbursement for their time and involvement. This research was approved by the institutional review committee of Sardar Bhagwan Singh University (SBSU) and was carried out in compliance with the Helsinki Declaration. The experiment was carried out at the physical therapy rehabilitation centers of the SBSU Dehradun, India. Only healthy participants were included in the study as there was a lack of evidence supporting the physio master application's validity and reliability. The same therapists and tools were used to evaluate each subject.

Instruments

Physio Master Application

**Figure 1:**
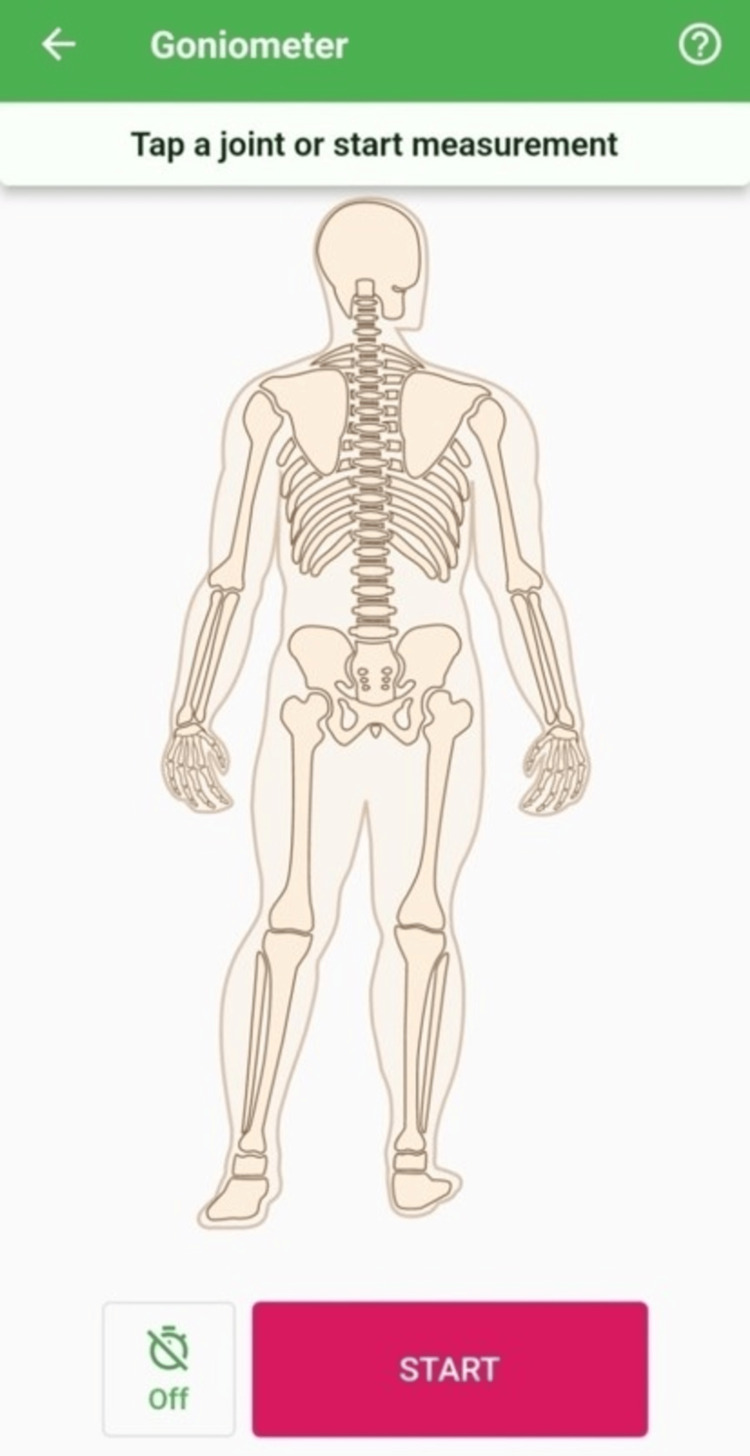
Android application for CROM CROM - Cervical Range of Motion

Physical therapists find this tool invaluable as it can be used for a variety of tasks, including evaluation of motion (angles, velocities, and accelerations), FMS analysis, accurate goniometer range of motion measurement, and ROM measurements on photographs. It can also store data separately for each patient that can be used as a database and is available for both iPhone and Android smartphones. Measurements of flexion and extension were obtained using the smartphone placed on the right external auditory meatus while the person was requested to flex and extend their neck. For the side-flexion on the opposite side of the head, the phone was positioned in line with the eyes, and for the cervical rotations the phone was placed over the vertex of the head aligned with the nose (Figure 1).

Digital Goniometer

**Figure 2:**
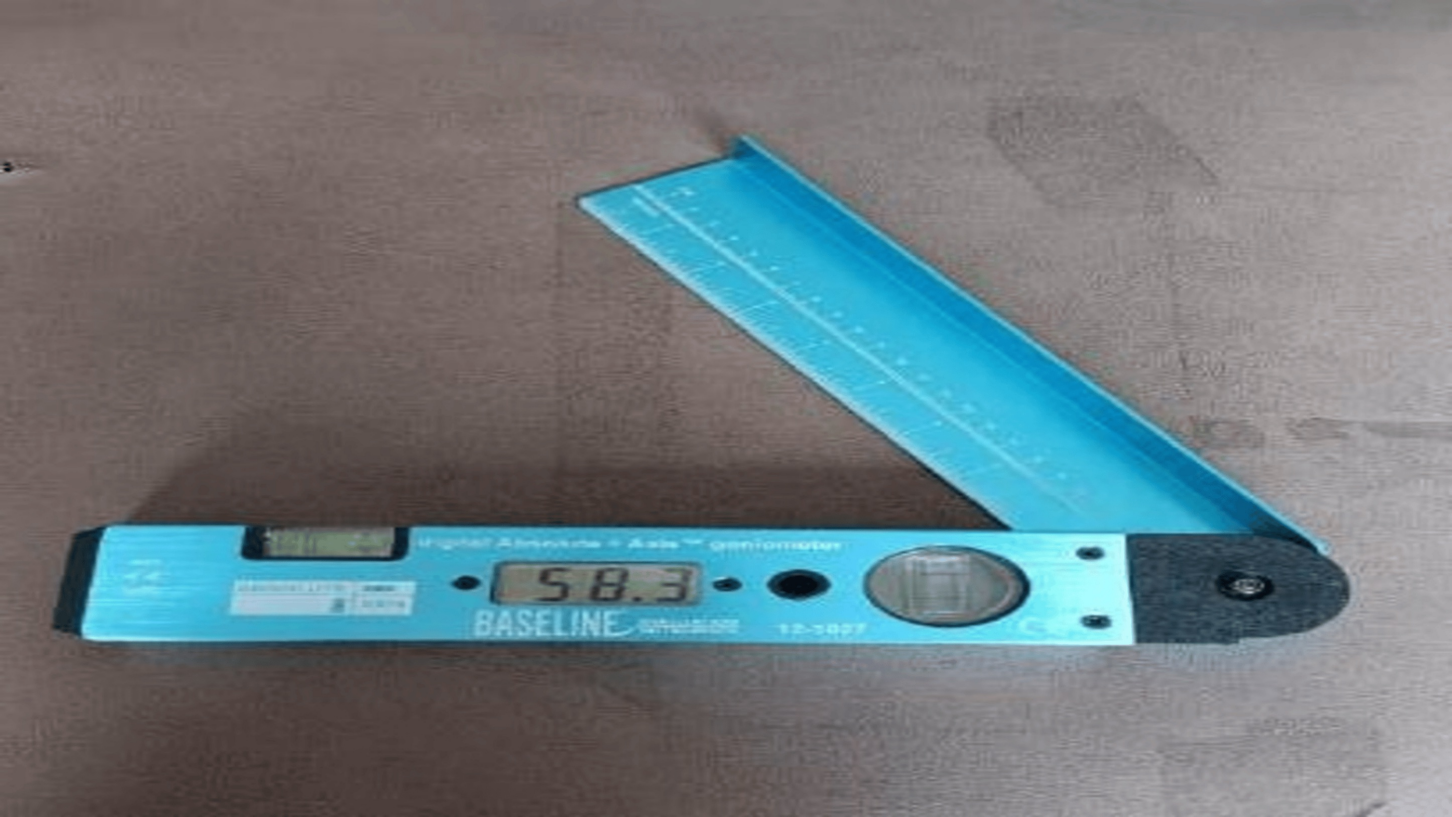
Digital goniometer

A baseline DG is used to measure the ROM of all the joints. The goniometer's axis for measuring neck extension and flexion on the acoustic meatus with the stationary arm upright and moving arm aligned with the nose (sagittal plane). The rotational axis was positioned at the vertex, with the stationary arm aligned with the side's acromion process and the moving arm aligned with the tip of the snout (planar horizontal). When performing rotations, the axis was positioned above the spinous process of the C7 vertebrae, the resting arm was aligned with the thoracic region's spinous process (frontal plane), and the movable arm followed the posterior centerline of the head (Figure 2).

Procedure

**Figure 3:**
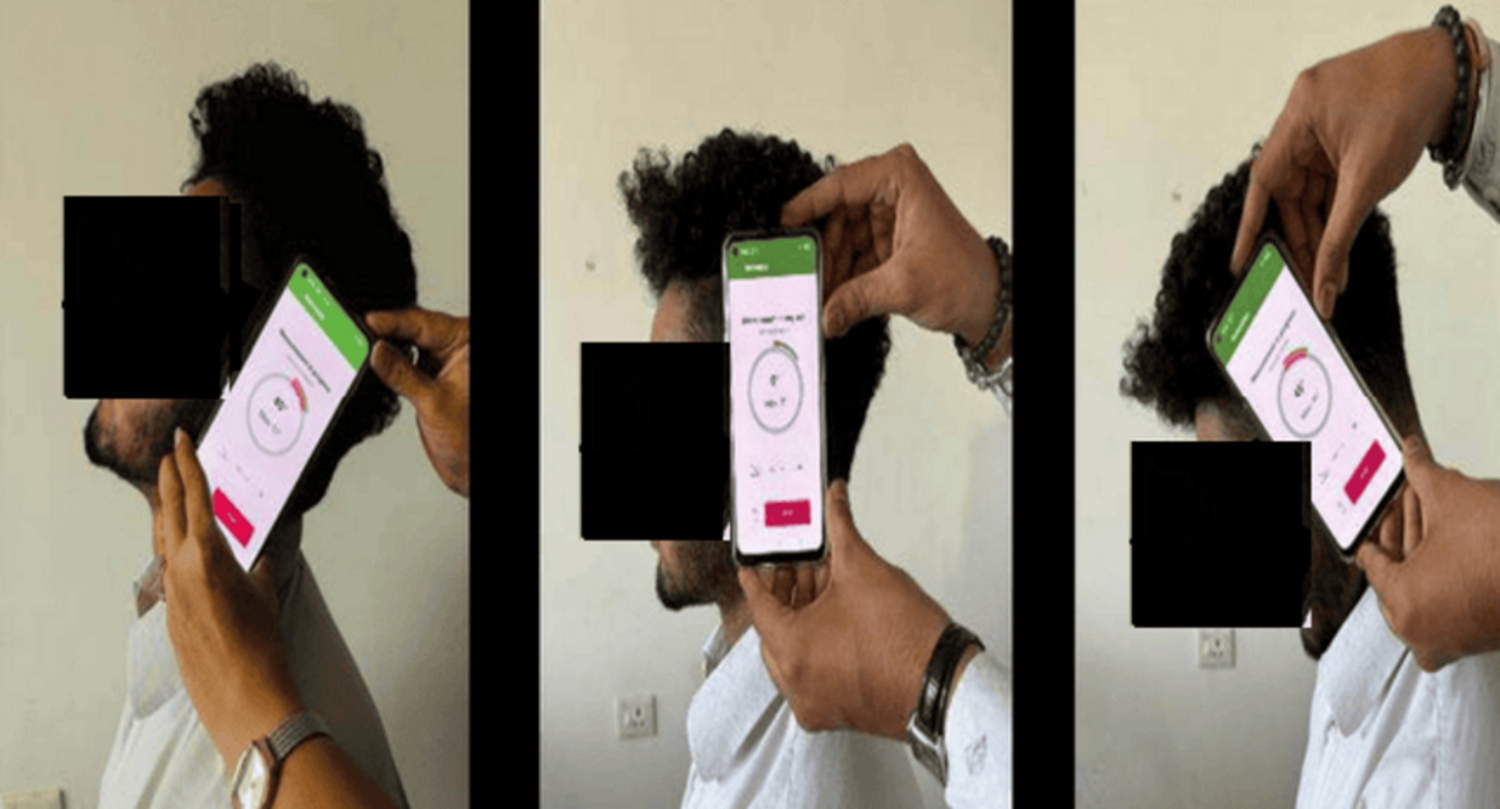
Position of smartphone for measuring flexion and extension

**Figure 4:**
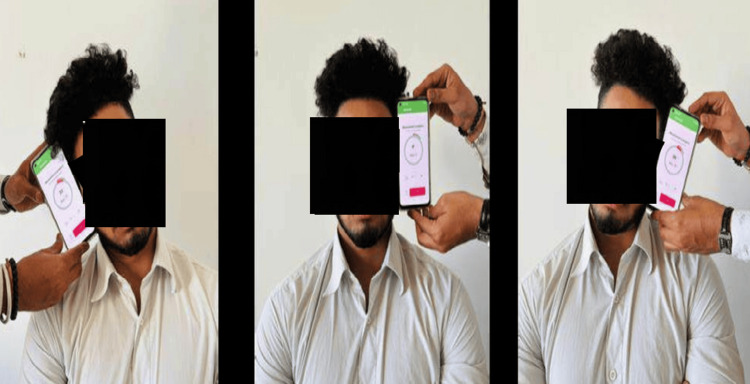
Position of smartphone for measuring lateral flexion

**Figure 5:**
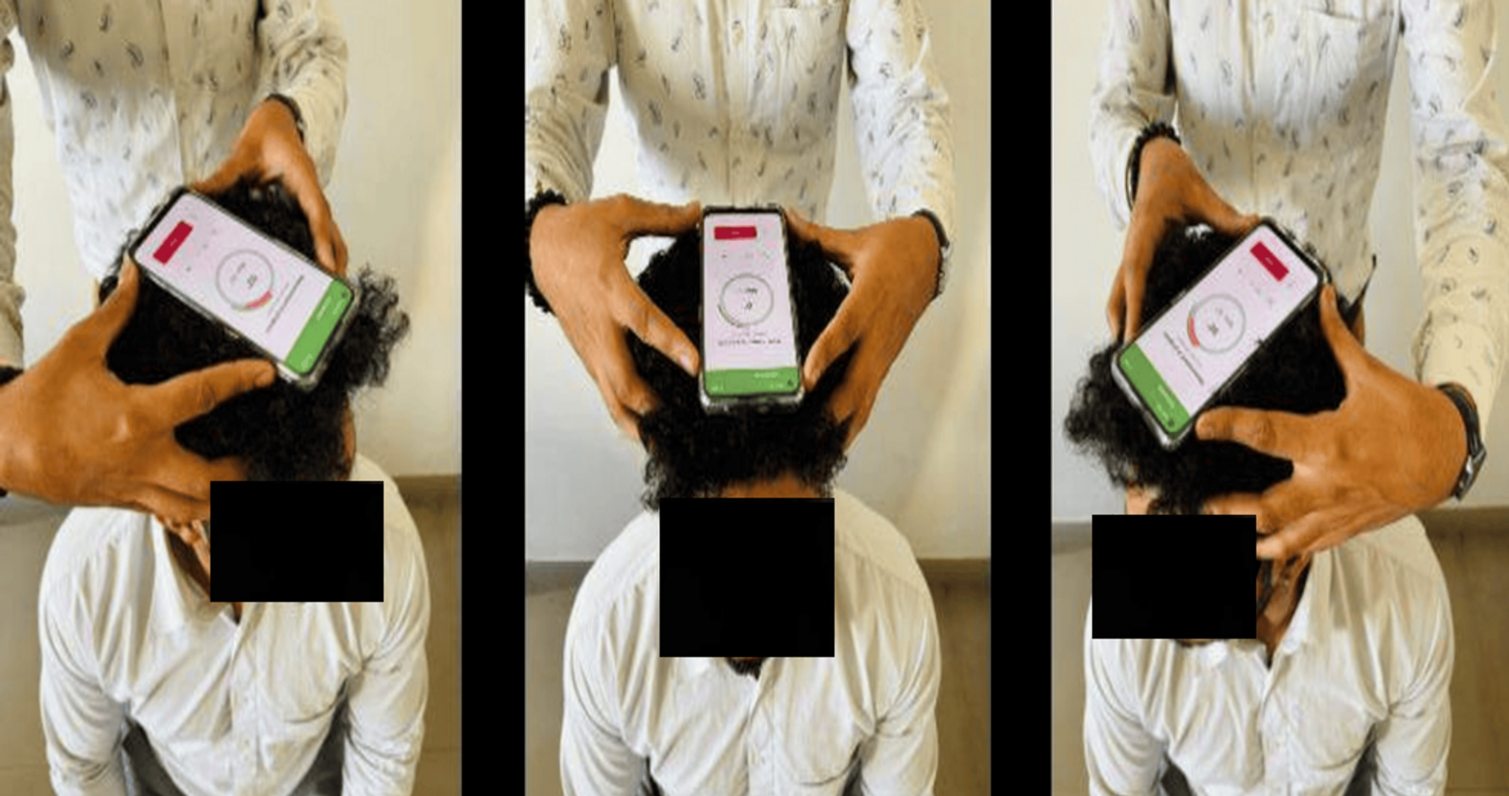
Position of smartphone for measuring rotation

Participants in this study were instructed to execute maximal (end-range) flexion, extension, and rotation movements on both the left and right sides. They were instructed to perform these movements at a natural pace, avoiding rapid execution. All participants performed these movements before the measurements to eliminate any restrictions. Four final-year physical therapy students were given three hours of instruction to use the application and DG properly. The subjects were informed about the technique used for data collection after which they performed each cervical movement five times. To minimize bias, the following movements were measured in sequence flexion, extension, side flexion (left and right), and rotation (right and left) (Figures 3-5).

Data collection

Procedure for Reliability Study

To conduct reliability research, two therapists independently entered two separate cabins, measured all cervical motions (including flexion, extension, and side flexion on the right and left, as well as left and right rotations) using a smartphone (one plus 9R), and recorded the results. After that, the therapists changed the rooms in a clockwise orientation until they had twice measured each movement using the smartphone application evaluating rater reliability within and between groups which was the aim of the study.

Procedures for Validity Study

**Figure 6:**
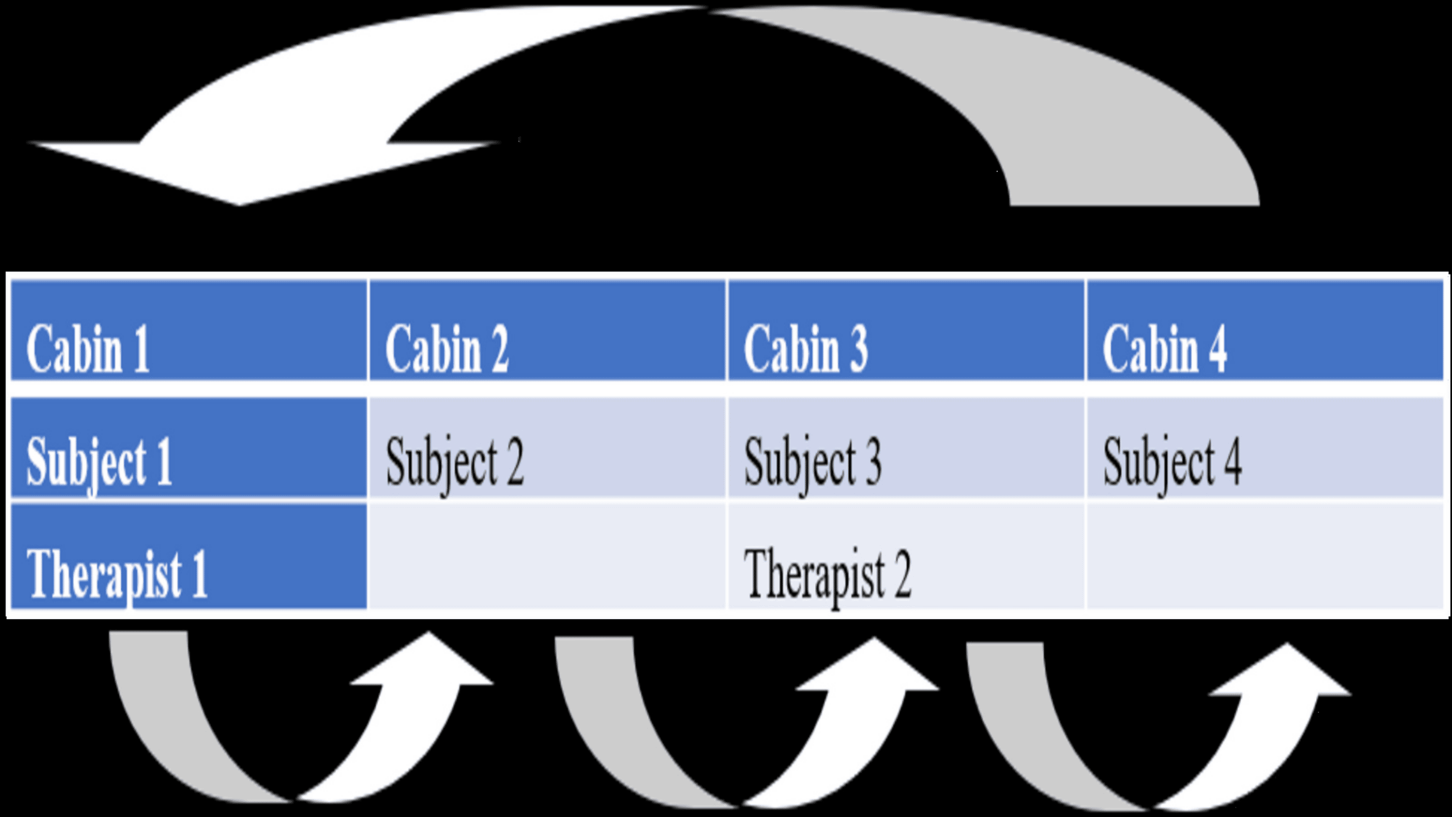
Data collection process

The first examiner entered room A and used the DG to measure every cervical movement. The examiner then moved from one room to another clockwise order and measured every patient once. This aided in assessing the physio master application's criterion validity in relation to the DG (Figure 6).

Data analysis

In the study's reliability section, the intraclass correlation factor (ICC) was used to measure intra-rater and inter-observer consistency. The magnitude and trend of the relationship between two variables can be determined using the ICC statistic [10]. Perfect positive association (+1) and perfect negative association (−1) are the range of values. A reasonable scale for interpreting ICC is as follows: an ICC value <0.4 indicates poor reproducibility; an ICC value in the middle of 0.4 and 0.75 indicates fair to good reproducibility; and an ICC value >0.75 indicates excellent reproducibility. Different guidelines exist for interpreting ICC. We applied the ICC of correlation to determine the criterion validity. When interpreting the internal consistency coefficients (ICCs) for the validity portion of the study, a value of < 0.5 denotes poor validity, a value in the middle of 0.5 and 0.65 indicates moderate to good validity, and a value >0.65 denotes good validity. The point estimated was then surrounded by 95% confidence intervals (95% CI) to account for sample variation. For both the smartphone application and the DG, descriptive statistics are provided using mean and standard deviation for measurements of ROM (degrees) for each movement.

Interpretation of Reference Values ICC

Reliability: Good: ≥ 0.75, moderate: 0.40 to 0.75, poor: ≤ 0.40

Validity: Good: ≥ 0.65, moderate: 0.50 to 0.65, poor: ≤ 0.50

## Results

Intra-rater reliability

The ICCs for the first therapist showed excellent reliability which varied from 0.77 to 0.96 where extension and left rotation showed the best ICCs 0.96 each, and flexion showed the lowest ICC 0.77. The ICCs for the second therapist also showed excellent reliability which varied from 0.86 to 0.93 where extension side flexion to the left showed the best ICCs of 0.93 and 0.91, respectively, and flexion showed the lowest ICC value of 0.86.

Inter-rater reliability

We analyzed the mean range of motion for each movement made by the two assessors to determine the inter-rater reliability. We found excellent inter-rater concordance varying between 0.86 and 0.96. Right side flexion displayed the lowest ICC of 0.86 and extension the highest, at 0.96. Flexion and left rotation showed ICCs of 0.87 each whereas right rotation and left side flexion showed ICCs of 0.89 and 0.9, respectively (Table 1).

**Table 1:** Mean and standard deviation of ROM of cervical movements in degrees

Movement	Mean and standard deviation (degrees)				
	Digital goniometer	Physio master application			
	Therapist 1	Therapist 1, Reading 1	Therapist 1, Reading 2	Therapist 2, Reading 1	Therapist 2, Reading 2
Flexion	40.20 ± 6.07	40.33 ± 7.09	40.93 ± 5.58	42.05 ± 6.14	41.84 ± 5.32
Extension	47.54 ± 8.75	49.46 ± 7.52	49.14 ± 7.61	50.7 ± 7.46	50.04 ± 7.30
Right rotation	59.93 ± 6.79	69.57 ± 6.27	63.53 ± 6.17	64.34 ± 6.05	63.66 ± 6.17
Left rotation	65.67 ± 9.01	68.81 ± 7.74	68.2 ± 7.34	67.83 ± 7.60	68.36 ± 7.06
Right side flexion	42.16 ± 4.85	41.75 ± 4.11	41.51 ± 4.23	40.63 ± 4.53	42.21 ± 3.70
Left side flexion	42.13 ± 4.96	41.48 ± 4.72	41.42 ± 4.24	41 ± 4.75	41.48 ± 4.09

Criterion validity

For criterion validity, we compared the reference standard DG with the average of the first therapist’s measurements. We found excellent validity with DG ICCs ranging between 0.79 and 0.93. Extension showed the best validity with the DG (ICC = 0.93) and right rotation showed the least validity with the gold standard (ICC = 0.79) flexion showed 0.86 while rotation left, side-flexion right and left showed 0.8, 0.82, and 0.81 ICCs, respectively (Table 2).

**Table 2:** Using physio master application, ICC for intra- and inter-observer reliability, and criterion validity

Movements	Intra-rater		Inter-rater	Criterion validity
	Therapist 1	Therapist 2		
Flexion	0.76	0.86	0.86	0.85
Extension	0.95	0.92	0.96	0.93
Right rotation	0.88	0.89	0.89	0.78
Left rotation	0.95	0.87	0.87	0.80
Right side flexion	0.85	0.89	0.86	0.81
Left side flexion	0.90	0.90	0.89	0.80

## Discussion

The study seeks to evaluate the within and between-rater reliability, and concrete validity of the physio master application in contrast to the standard DG. Although there have been studies on the validity and reliability of a range of motion applications, no previous study has evaluated the validity and reliability of physio master application in measuring cervical range. Since there was no prior research on the physio master application, we included only healthy participants to eliminate any potential bias.

An instrument must be reliable to be deemed valid; reliability estimates are crucial psychometric qualities. The present study showed excellent intra-rater reliability for both therapists in all the movements of cervical ROM. Therapist 1 showed the maximum ICCs in extension and left rotation, i.e. 0.96, and flexion showed the lowest ICC value 0.77. Whereas therapist 2 showed maximum ICC for the extension, i.e. 0.92, and least ICC for flexion, i.e. 0.86. When the results of two examiners were compared our ICCs were excellent for extension which was 0.96 and poor for right side flexion which was ICCs 0.86.

For the criterion validity when we compared the physio master application with reference standard DG. These DGs are quick and simple to use, which lowers the possibility of error, according to Norkin. Nevertheless, there are certain drawbacks to using the DG: it is expensive, needs two hands to operate, can be difficult to align, and requires clear visual judgment for measurement reading. Measurement errors may occur due to these restrictions [11].

We found excellent validity, especially in extension with ICC = 0.93. However, poor results were obtained for the right rotation with ICC = 0.79. The fact that it was recorded by a tool very sensitive to electromagnetic fields may help to explain this in part. This may cause the measurement's accuracy to decline. It might also be explained by how the smartphone was held and/or moved while the cervical rotation was being measured.

As far as we are aware, no research has been released that looks at the applicability of the Physio master for measuring neck ranges. To determine neck range of movement in healthy individuals, Tousignant-Laflamme et al. published a paper on the criterion validity and reliability of two iPhone applications. The study's findings, however, indicated poor inter-rater consistency (ICC < 0.60) and moderate intra-rater reliability (ICC = 0.65-0.85) for each movement. For flexion, extension, lateral flexions, and right rotation, the ICCs for the criterion validity range from moderate (>0.50) to good (>0.65), whereas for left rotation, they are poor (0.50) [12].

There is another study performed by Steven et al., this is an investigation that checks the validity of simultaneously measuring knee range of motion with a regular UG and an application for measuring knee ranges to assess intra- and inter- measurer reliability of inexperienced and seasoned doctors. The study found a high level of agreement between measurements taken with a smartphone goniometric application (SGA) and a UG, with average concordance correlation coefficients (CCC) above 0.96 for each examiner. The UG had a Standard Error of Measurement (SEM) of 1.56° (ranging from 0.52° to 2.66°), while the SGA had an SEM of 0.62° (ranging from 0.29° to 1.27°). Both devices consistently provided accurate measurements of knee flexion angles [5].

Limitations of the study

The external validity of the research is constrained by the focus on cervical ranges and the inclusion of only healthy participants. Moreover, external validity could be affected by the fact that the conditions under which the measurements were taken (such as the location of the participants) may not accurately represent real-life settings. Before the measurements were taken, participants performed a warm-up by repeating all cervical movements, which might have influenced the results due to possible fatigue or increased flexibility. Even though the examiners were trained to reduce measurement errors, any remaining variations in their skill or interpretation of the measurement method could influence the overall outcomes. Neck ranges were assessed by a variety of techniques (ROM). The study, for instance, made use of a smartphone application that showed low validity, particularly for rotational movements, but moderate to good validity for other movements. The constancy of the smartphone as a measurement tool in all directions is called into question by this inconsistency.

## Conclusions

The study confirms that both intra and inter-observer reliability, as well as concrete validity, of the physio master application are strong. This enhances its potential use in physiotherapy practices, particularly in evaluating and monitoring cervical disorders. The research contributes insightful information on determining the cervical range of motion and emphasizes the significance of employing trustworthy measurement instruments in real-world settings.
